# 

**Published:** 1913-05-15

**Authors:** 


### ANNOUNCEMENTS.

Ohio State Dental Society. Preliminary notice of the Ohio State Dental Society meeting to be held at Toledo, December the 2d, 3d and 4th, 1913. This meeting will be an innovation. It will mark the beginning of a new epoch in state dental meetings.

Nowhere at any time has there been planned a meeting like this. Fields heretofore untouched will be opened at this meeting. Men of national and international reputation will be in attendance and present subjects along the lines of scientific research, systemic treatment and preventive measures of incalculable value to the dental profession.

The Clinics will also mark a new epoch—only fifteen clinicians, men who have attained the highest proficiency in their respective fields, will be here. The unit system will be used and every dentist in attendance will HEAR and SEE all that each clinician says and does.

Toledo is on the main line between New York and Chicago, and has the finest restaurants, hotels and theatres between these two great centers. Toledo is known as the Golden Rule city. Our police carry no clubs—they apply the golden rule. Come and see it in operation. No dentist need stay away on account of the police.

The Commerce Club, occupying the two upper top floors, of the 16-story Nicholas building has graciously extended the privileges of the club to visiting dentists.

Exhibitors desiring to secure space, and all others interested should address

COMMITTEE LOCAL ARRANGEMENTS,
718 SPITZER BUILDING.

---

WEST VIRGINIA STATE SOCIETY. The seventh annual meeting of the West Virginia State Dental Society will be held in the Assembly Room of the Chancellor Hotel, Parkersburg, W. Va., August 13, 14, 15, 1913. Opening session, 2 o'clock, Wednesday, August 13. A cordial invitation is extended to all ethical members of the profession to attend our meeting.

FRANK L. WRIGHT, *Secretary.*

---

NORTHERN INDIANA DENTAL SOCIETY. The annual meeting of this society will be held at Gary, Ind., September 23—25. Preparations are being made for an excellent meeting in this new and interesting city and a cordial invitation is extended to all dentists to attend.

W. LEROY MYER, *Secretary,*
Rensselaer, Ind.

---

NORTHERN OHIO DENTAL ASSOCIATION. The annual meeting of the Northern Ohio Dental Association will be held at the Hollenden Hotel, Cleveland, Ohio, Thursday, Friday and Saturday, June 5, 6 and 7, 1913.

The executive committee has arranged a program which they feel will meet the approval of all who attend.

We do not believe it necessary to make any mention of Cleveland's advantages as a convention city, as everyone is familiar with the sixth city and its numerous attractions and accommodations.

A little later you will receive more information and also the program.

Mark off the above dates and be sure to attend this meeting.

J. K. DOUGLAS, *Chairman.*
J. M. YAHRES,
W. H. VAN DEMAN.

MINNESOTA STATE DENTAL ASSOCIATION. The thirtieth annual meeting of the Minnesota State Dental Association will occur in Masonic Temple, Minneapolis, Friday and Saturday, June 13 and 14, 1913.

Every phase of dentistry will be presented by men of unquestioned ability from Dr. Roper of Indianapolis with steriopticon lexture on treatment of root canals, to the effect of pathological conditions of the mouth on systemic conditions which will be presented by Dr. C. H. Mayo of Rochester, whose name is familiar wherever medicine is practiced. A large manufacturers exhibit will round out what we expect to be an unusally large meeting of the organization.

BENJAMIN SANDY, *Secy.*,
Syndicate Block, Minneapolis.

MICHIGAN STATE BOARD OF EXAMINERS. The next regular meeting of the Michigan State Board of Dental Examiners will be held at the Dental College, Ann Arbor, commencing Monday, June 16th, at 8 a. m., and continuing through the 21st. For application blanks and full particulars address

F. E. SHARP, *Sec'y*,
Port Huron, Mich.

JUNE MEETINGS: American Society of Orthodontists, June 30th to July 2nd, at Chicago.

California State Meeting, June 2nd to 5th, 1913, at Oakland.

Colorado State Meeting, June 19th to 21st, at Manitou.

Georgia State Association, June 12th to 14th, at Columbus.

Mississippi State Association, June 24th to 26th, at Meridian.

Minnesota State Association, June 13th and 14th, at Minneapolis.

Michigan State Board of Examiners, June 16th to 21st, at Ann Arbor.

Indiana State Board of Examiners, June 9th to 14th, at Indianapolis.

Illinois State Board of Examiners, June 4th, at Chicago.

Pennsylvaia Examiners, June 11th to 14th at Philadelphia and Pittsburgh.

Ohio Board of Examiners, June 17th, at Columbus.

West Virginia Board of Examiners, Wednesday June 11th at Huntington.

Tennessee State Dental Association, June 5th to 7th, at Nashville.

Northern Ohio Dental Association, June 5th to 7th, at Cleveland.

Northern Michigan Dental Society, June 6th and 7th, at Houghton.

Wisconsin Board of Examiners, June 16th, at Milwaukee.

TENNESSEE STATE DENTAL ASSOCIATION. The next meeting of the Association will be held on the 5th, 6th and 7th of June in Nashville, Tenn. A carefully prepared program insures a good meeting and all ethical dentists will be welcomed.

C. OSBORN RHEA, *Sec'y.*

NATIONAL DENTAL ASSOCIATION. The 1913 session of the National Dental Association will be held in Kansas City, Mo., July 8th to 11th. The re-organization of the Association should make this the most important meeting in its history. Every State Society that has met since the new Constitution and By-Laws were adopted, at the Washington meeting, has voted to become a constituent society, and we can all appreciate the influence of such an organization if all the State Societies take similar action.

The officers and committees have been active in preparing an exceptionally interesting program. At this date the following literary program is tentatively announced:

Dr. Frank O. Hetrick, Ottawa, Kan., "President's Address"; Dr. Adolph Fenchel, Hamburg, Germany (subject to be announced later); Dr. Weston A. Price, Cleveland, O., "Scientific Foundation Fund"; Dr. Roscoe A. Day, San Francisco, Cal., "Orthodontia and its Relation to Dentistry"; Dr. Marcus L. Ward, Ann Arbor, Mich., "Metallurgy"; Dr. Richard L. Simpson, Richmond, Va., "Unbanded vs. Banded Crowns"; Dr. Percy H. Howe, Boston, Mass., "The Saliva"; Dr. Arthur D. Black, Chicago, Ills., "Something of the Etiology and Early Pathology of Diseases of the Peridental Membrane, with Suggestions as to Treatment"; Dr. Hermann Prinz, Philadelphia, Pa., "A Preliminary Report on Action of $As_2O_3$"; Dr. Howard R. Raper, Indianapolis, Ind., "The Value of the Radiograph in the Practice of Modern Dentistry"; Dr. G. S. Junkerman, Cincinnati, O., "Dental Educational Harmony"; Dr. Clarence J. Grieves, Baltimore, Md., "Periapical Infections"; Dr. J. J. Sarrazin, New Orleans, La., "Prophylaxis"; Dr. H. B. Tileston, Louisville, Ky., "Diagnosis and Treatment of Diseases of the Dental Pulp."

The Clinic Committee has been very energetic in preparing their program and we have every reason to expect that they will present a very strong list of clinicians. The Local Committee of arrangements are providing ample facilities for a large meeting and have selected the Baltimore Hotel as headquarters. We are not prepared at this time to publish a list of hotels and their rates, nor to incorporate anything pertaining to railroad rates. Those desiring to make hotel reservations in advance should address the Baltimore Hotel, or the Chairman of Local Committee of Arrangements, Dr. Charles C. Allen, No. 507 Rialto Bldg., Kansas City, Mo. Information regarding transportation from any point may be secured from any local railroad agent, as they have full information with reference to all rates granted by various passenger associations in their particular territory.

All reputable practitioners of Dentistry and Medicine are cordially invited to attend this meeting.

Fraternally,

FRANK O. HETRICK, *President*, Ottawa, Kans.
HOMER C. BROWN, *Rec. Sec'y*,
185 East State St., Columbus, Ohio.

### KANSAS CITY HOTEL INFORMATION.

| | | | | |
|---|---|---|---|---|
| HOTEL BALTIMORE (Headquarters): | | | | |
| One person—without bath | $1.50 | and | $3.00 | per day |
| One person—with bath | 2.50 | " | 6.00 | " " |
| Two persons—without bath | 2.50 | " | 3.00 | " " |
| Two persons—with bath | 4.00 | " | 7.00 | " " |
| SEXTON HOTEL: | | | | |
| One person—without bath | $1.00 | per day and up | | |
| One person—with bath | 2.00 | " " " " | | |
| THE COATS HOUSE: | | | | |
| One person—without bath | $1.00 | to | $2.00 | per day |
| One person—with bath | 1.50 | " | 3.50 | " " |
| HOTEL SAVOY: | | | | |
| One person—without bath | $1.00 | to | $1.50 | per day |
| One person—with bath | 1.50 | " | 2.50 | " " |
| HOTEL VICTORIA: | | | | |
| Single—without bath | $1.00 | and | $1.25 | per day |
| Double—without bath | 1.50 | " | 2.00 | " " |
| Single—with bath | 1.25 | " | 1.50 | " " |
| Double—with bath | 2.00 | " | 2.50 | " " |
| HOTEL KUPPER: | | | | |
| Single—without bath | $1.00 | and | $1.50 | per day |
| Double—without bath | 2.00 | " | 2.50 | " " |
| Single—with bath | 1.50 | " | 2.50 | " " |
| Double—with bath | 2.50 | " | 4.50 | " " |
| DENSMORE HOTEL: | | | | |
| Single—without bath | $1.00 | per day | | |
| Double—without bath | 1.50 | " " | | |
| Single—with bath | 1.50 | " " | | |
| Double—with bath | 2.00 | to | $2.50 | per day |
| HOTEL EDWARD—Rates | $1.00 | per day and up | | |
| HOTEL WHITE: | | | | |
| Rooms—running water—hot and cold | $1.00 | per day | | |
| " shower or tub bath | 1.50 | " " | | |
| " outside—bath with tub | 2.00 | " " | | |

The above hotel information was received after our general notice was sent to the Journals. Would suggest that persons interested make reservation direct with the hotel of their selection. HOMER C. BROWN, *Rec. Sec'y.*

